# Mitochondrial Sirtuins in Reproduction

**DOI:** 10.3390/antiox10071047

**Published:** 2021-06-29

**Authors:** Giovanna Di Emidio, Stefano Falone, Paolo Giovanni Artini, Fernanda Amicarelli, Anna Maria D’Alessandro, Carla Tatone

**Affiliations:** 1 Department of Life, Health and Environmental Sciences, University of L’Aquila, 67100 L’Aquila, Italy; stefano.falone@univaq.it (S.F.); fernanda.amicarelli@univaq.it (F.A.); annamaria.dalessandro@univaq.it (A.M.D.); carla.tatone@univaq.it (C.T.); 2 Department of Obstetrics and Gynecology “P. Fioretti”, University of Pisa, 56126 Pisa, Italy; pg.artini@gmail.com

**Keywords:** mitochondria, mitochondrial sirtuins (mtSIRTs), SIRT3, SIRT4, SIRT5, ovary, testis, oocyte, sperm, fertility

## Abstract

Mitochondria act as hubs of numerous metabolic pathways. Mitochondrial dysfunctions contribute to altering the redox balance and predispose to aging and metabolic alterations. The sirtuin family is composed of seven members and three of them, SIRT3-5, are housed in mitochondria. They catalyze NAD+-dependent deacylation and the ADP-ribosylation of mitochondrial proteins, thereby modulating gene expression and activities of enzymes involved in oxidative metabolism and stress responses. In this context, mitochondrial sirtuins (mtSIRTs) act in synergistic or antagonistic manners to protect from aging and aging-related metabolic abnormalities. In this review, we focus on the role of mtSIRTs in the biological competence of reproductive cells, organs, and embryos. Most studies are focused on SIRT3 in female reproduction, providing evidence that SIRT3 improves the competence of oocytes in humans and animal models. Moreover, SIRT3 protects oocytes, early embryos, and ovaries against stress conditions. The relationship between derangement of SIRT3 signaling and the imbalance of ROS and antioxidant defenses in testes has also been demonstrated. Very little is known about SIRT4 and SIRT5 functions in the reproductive system. The final goal of this work is to understand whether sirtuin-based signaling may be taken into account as potential targets for therapeutic applications in female and male infertility.

## 1. Introduction

The recent increase of infertility worldwide is due to the unhealthy lifestyles, reduced exercise, and aging in modern society. This has led to growing interest in the role of energy metabolism in the maintenance of fertility potential. The mitochondria act as hubs of numerous metabolic pathways such as tricarboxylic acid cycle (TCA), β-oxidation of fatty acids, urea cycle, and pyrimidine nucleotide biosynthesis. Mitochondrial dysfunctions contribute to altering the redox balance and metabolic adaptation capacity, predisposing to aging and metabolic alterations. Healthy mitochondria are essential for mammalian reproduction, as stressed by the fact that mitochondrial dysfunction has been linked to subfertility and infertility at distinct levels [1,2,3,4]. Indeed, they are involved in numerous processes, such as steroidogenesis, apoptosis, homeostasis, and cell division, and are the primary source of ATP synthesis by oxidative phosphorylation (OXPHOS) [5,6]. About 85–90% of the oxygen within the cell is consumed by mitochondria during OXPHOS. Apart from ATP production, the intense oxidative metabolism in mitochondria is also associated with Reactive Oxygen Species (ROS) production [7]. ROS can function as mediators of cell signaling, but if their production is not controlled and balanced, ROS may act as undesirable extra and induce oxidative damage. About 0.2–2% of the oxygen taken up by the cells is converted to ROS by mitochondria. To cope with oxidative damage, mitochondria are also provided with various low-molecular-weight antioxidants, as well as by multiple enzymatic defense systems [5].

The role of mitochondria in reproduction, and particularly the biological competence of gametes, has emerged in recent years so opening new frontiers in the study of infertility [1,2,3,4]. Mammalian oocytes are long-lived cells. They initiate meiosis in the embryonic ovary, arrest meiotically for long periods and resume meiosis at time of ovulation after an extensive growth phase. The number and aspect of mitochondria change during oogenesis and early stages of embryo development [2]. Metaphase II stage oocyte possesses between 150,000 and 300,000 mitochondria, a condition indicative of the energetic cell demand [2,4]. Dysfunctional mitochondria are a major factor in the predisposition to aberrant development of embryos after fertilization. Oocyte mitochondria are essential for early embryonic development. Paternal mitochondria are crucial to sperm function and fertilization. Indeed, mammals inherit their mtDNA from the population present in the oocyte just prior to fertilization when mtDNA replication has been completed [8,9]. Following natural fertilization, sperm mitochondria, and thus also mtDNA are eliminated prior to embryonic gene activation [1]. Mitochondria in spermatozoa are at the center of the metabolism and are involved in the redox balance, calcium regulation and apoptotic pathways, which are necessary for their fertilization potential. Their deterioration has been linked to an alteration in sperm parameters and/or fertility [3,10]. Therefore, given the central role played by mitochondria influencing germ cell functions, it appears clear that their regulation in terms of number and functionality is essential [1,2,3,4,9,10].

Over the decades, factors intrinsic and extrinsic to mitochondria have been found to ensure an efficient metabolic flow and an appropriate energy balance. Among the major mitochondrial regulators, NAD-dependent deacetylases of the sirtuin family have emerged to play an important role by their direct and indirect influence. Mammals possess seven sirtuin paralogs (SIRT1-7) with divergent terminal primary structures responsible for different subcellular localizations. Since sirtuin function is essentially dependent on the NAD+/NADH ratio, these protein families may serve as sensors of the cellular energy status. This may be particularly important within mitochondria, which host a high number of acylated proteins and contain high levels of NAD+ and NADH [11]. Differences in the sub-cellular localization of sirtuins consent spatially varying control of protein post-translational modifications (PTMs). SIRT3-5 have a mitochondrial localization and are believed to serve as important factors that coordinate key aspects of mitochondrial function.

The present review represents the first attempt to summarize current knowledge on mitochondrial sirtuins (mtSIRTs) in reproduction. Our aim was to provide challenges and opportunities to stimulate research on the role of mtSIRTs in the regulation of mitochondrial functions and redox homeostasis in mammalian oocytes, sperm, embryos, ovary and testis in relation to preservation of fertility potential.

## 2. Mitochondrial Sirtuins

**Figure 1:**
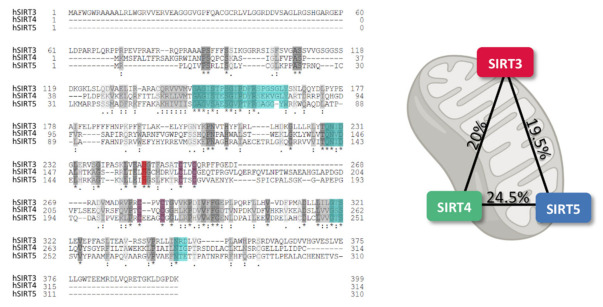
Multiple alignment with sequence similarities (grey), nucleotide binding sites (cyan), zinc-binding residues (purple), and active sites (red), shared by human mtSIRTs (hSIRT3-5) (left panel), and identity percentages among hSIRT3-5, as calculated by Clustalo (right panel).

SIRT3-5 have been found mainly within the mitochondrion, although some researchers have also reported extra-mitochondrial localization [12,13]. The precise molecular mechanisms underlying subcellular transports of mtSIRTs remain poorly understood, however, the presence of N-terminal mitochondrial targeting sequence (MTS) allows mtSIRTs to localize within the mitochondrial matrix [14,15]. mtSIRTs are zinc-dependent enzymes that share common metal binding residues and conserved N-terminal nucleotide binding domains, with approx. 10% sequence identity shown in humans (Figure 1). However, SIRT4 and SIRT5 exhibit much more overlapping regions [16]. How different primary structures reflect on the diverse functions of mtSIRTs remains to be fully elucidated. mtSIRTs crosstalk appears to be essential to define a molecular landscape in physiology and organize a coordinated stress response [17,18,19,20].

Until 2007, little was known about the biological functions of mtSIRTs [21], however, today we know that mtSIRTs are regulators of metabolic homeostasis by promoting PTMs of many protein targets [14,22]. Interestingly, the activity and expression of mtSIRTs are regulated by transcriptional and post-transcriptional mechanisms, including miRNAs [23].

Well beyond their well-characterized histone deacetylase activity, SIRTs catalyze a wide range of NAD+-dependent reactions, and these reactions target several non-histonic proteins. Importantly, mtSIRTs catalyze deac(et)ylation reactions, whereas SIRT4 shows a peculiar ADP-ribosyl transferase activity [24].

SIRT3 was the first mtSIRT found in mammals [25]. SIRT3 was initially found to deacetylate the metabolic enzyme glutamate dehydrogenase (GDH), thus promoting the oxidation of amino acids [26,27]. SIRT3 has been also described as a promoter of ketogenesis via activation of 3-hydroxy-3-methylglutaryl CoA synthase-2 (HMGCS2) [28,29]. Convincing evidence suggests that SIRT3 also promotes both mitochondrial respiration and OXPHOS. In fact, SIRT3 deacetylates all the electron transport chain (ETC) complexes, thus facilitating the electron flow and maximizing ATP production [14,30,31,32,33,34,35]. Moreover, SIRT3 deacetylates and activates pyruvate dehydrogenase (PDH), thus promoting a metabolic switch from anaerobic glycolysis to aerobic metabolism [36]. Also, it has been recently shown that the SIRT3-dependent enhancement of aerobic oxidation strictly involves the activation of peroxisome proliferator-activated receptor gamma coactivator 1-alpha (PGC-1α) and 5′ AMP-activated protein kinase (AMPK), thus implying mitochondrial biogenesis [37]. In addition, SIRT3 positively regulates the β-oxidation of fatty acids (FAO) through the deacetylation of acetyl-CoA synthetase 2 (AceCS2) and long-chain acyl-CoA dehydrogenase (LCAD) [38]. Moreover, SIRT3 controls amino acid utilization, as well as the normal progression of the Krebs cycle, by activating GDH and isocitrate dehydrogenase 2 (ICDH2) [39]. Interestingly, a quite recent proteomics investigation corroborated the notion that SIRT3 serves as a regulator for amino acid metabolism, FAO, and the regulation of TCA cycle and ETC [20].

Interestingly, by enhancing GDH and ICDH2 activities, SIRT3 helps cells to maintain adequate levels of NADPH. This high-energy electron donor is essential to the recycling of glutathione (GSH), which is required as cofactor by several crucial antioxidant enzymes [9]. This gains importance as mitochondria are a notable source of ROS, which when exceeding physiological levels can lead to chemical modification of macromolecules, thus leading to cell dysfunction and, ultimately, cell death [40]. SIRT3 controls the mitochondrial antioxidant milieu also by deacetylating and activating manganese-dependent superoxide dismutase (SOD2; a.k.a. MnSOD), the first-line scavenger for ETC-generated O2 [41,42,43,44]. Importantly, SOD2 (together with other antioxidant enzymes, like catalase, CAT) is also activated by SIRT3 via FoxO3a, a core transcriptional regulator of cellular homeostasis and stress response [45]. Interestingly, not only can SIRT3 limit the amount of mitochondrial ROS (mtROS), but it also promotes the repair of oxidative DNA damage, especially in mitochondrial DNA (mtDNA), via the stabilization of 8-oxoguanine-DNA glycosylase 1 (OGG1), which repairs oxidative damage to DNA [46]. In coherence with the antioxidant effect of the pathways activated by SIRT3, this protein is currently considered as a key factor that assists cells and stem cells during differentiation, a process that is often paralleled by mitochondrial activation and ROS overproduction [47]. Finally, in 2007, Prof. David A. Sinclair’s team showed that SIRT3 protects mitochondria from genotoxic stress-induced depletion of NAD+ [21], thus strengthening the idea of a crucial role of SIRT3 in maintaining mitochondrial balance.

The uncontrolled buildup of mtROS causes extensive and generalized protein damage, which if not cleared by re-folding and/or proteolysis, may lead to dysfunctional mitochondria [48]. SIRT3 has recently been suggested to regulate mitochondrial quality control (mtQC), thus having a role in the molecular network that prevents the accumulation of damaged mitochondria. In fact, SIRT3 seems to serve as an activator of the mitochondrial unfolded protein response (mtUPR), which involves changes in mitochondrial fusion and fission, and, ultimately, in the selective removal of dysfunctional mitochondria by mitophagy [49]. More in depth, proteolysis targets mitochondrial oxidized proteins thanks to the Lon protease, and SIRT3 has been found to activate Lon by deacetylation [50]. Mitochondrial dynamics is also regulated by SIRT3. On one hand, SIRT3 prevents mitochondrial division and ATP shortage via deacetylation of the fusion protein GTPase optic atrophy 1 protein (OPA1), on the other hand SIRT3 promotes the FoxO3-dependent increase in the expression of the pro-fusion protein mitofusin 2 (MFN2) [51,52]. Furthermore, SIRT3 acts as a crucial mediator of the mitophagic response to oxidative stress (OS)-induced mitochondrial damage, and this seems to be linked to the activation of the FoxO3A-Pink1-Parkin axis [53,54,55,56,57]. Of note, most of these studies were conducted on models of neurodegenerations. SIRT3 protects mitochondria also by preventing the assembly of the mitochondrial permeability transition pore (mPTP), whose formation facilitates energy imbalance and apoptotic death via disruption of the transmembrane proton gradient [58].

To summarize, the literature suggests that SIRT3 regulates metabolic balance enhancing energy production in mitochondria, promoting metabolic flexibility in conditions of changes of energy requirements. Moreover, SIRT3 participates to the molecular system that warrants adequate levels of cytoprotection against redox endogenous and exogenous stressors, in physiological and pathological conditions. Furthermore, recent evidence pointed to SIRT3 as a critical regulator of mitochondrial quality control via the modulation of mitochondrial proteostasis, dynamics, and autophagic removal.

Other possible functions of SIRT3 have been suggested. For example, SIRT3 has been reported to promote microtubule turnover, generating the pulling force needed for chromosome separation [59]. Moreover, a very recent work revealed a novel role for SIRT3 in stabilizing heterochromatin and counteracting the senescence process in human mesenchymal stem cells [60].

Among the mtSIRTs, SIRT4 is probably the most puzzling one, mainly due to the scarcity of well-documented enzymatic activities. In addition to the deac(et)ylase activity of the other sirtuins, SIRT4 exhibits a peculiar ADP-ribosyl transferase activity, that consists in transferring the ADP-ribose moiety from NAD+ to the target protein [61].

Despite the relatively recent investigations, some insights into the mechanisms of SIRT4-dependent regulation of key biological functions have emerged. The mitochondrial localization of SIRT4 and the abundance of SIRT4 in metabolically active organs raised the hypothesis that metabolic proteins might be targeted by SIRT4 [61]. The role of SIRT4 in the regulation of mitochondrial function was first demonstrated fifteen years ago by Prof. Leonard Guarente’s team [62]. This pioneering paper showed that SIRT4 is able to post-translationally inactivate mitochondrial GDH, linking SIRT4 to the control of pancreatic β cell function and amino acid-stimulated insulin secretion [62,63]. In fact, by ADP-ribosylating GDH SIRT4 prevents glutamine from serving as an insulin secretagogue in β cells [62]. Others showed that SIRT4 reduced via lipoamidation the activity of PDH, which governs the entrance of acetyl-CoA into the TCA cycle [64], thus reinforcing the idea that mitochondrial metabolism, along with the source of carbons for the mitochondrial oxidative catabolism, are profoundly influenced by SIRT4.

Beyond its regulating role in oxidative metabolism, SIRT4 seems to control ATP homeostasis directly. Accordingly, deletion of SIRT4 results in reduced ATP levels, whereas overexpression of SIRT4 increases ATP concentrations [14,56]. In particular, acylation of ATP/ADP translocase 2 (ANT2) in the inner mitochondrial membrane (IMM) is associated with mitochondrial uncoupling, hence SIRT4-dependent deacylation of ANT2 would reduce mitochondrial uncoupling, thus increasing ATP production [65]. In 2013, an acetylome microarray-based study identified several physiological candidates as deacetylation substrates for SIRT4. Among those targets, researchers found mtHsp60 and Stress-70, both implicated in cell proliferation and aging, as well as NAD(P) transhydrogenase, an enzyme important to direct redox-related stress responses [66], thus corroborating the hypothesis that SIRT4 regulates cell metabolism also via its neglected deacetylating function. This was later confirmed by Laurent and co-workers, who showed that SIRT4 deacetylated and inhibited malonyl CoA decarboxylase (MCD) [67], thus preventing the conversion of malonyl-CoA into acetyl-CoA. By favoring the build-up of malonyl-CoA, SIRT4 simultaneously reduces both fatty acid biosynthesis and β-oxidation within the mitochondrion [68]. In addition, in 2016, some researchers demonstrated that the mitochondrial trifunctional protein α-subunit (MTPα), a critical FAO-promoting enzyme, is deacetylated and destabilized by SIRT4 [69]. All these observations suggests that SIRT4 is an important negative regulator of FAO and biosynthesis processes [70].

SIRT4 also participates to the complex mechanism by which cell controls the redox milieu. In 2007, Prof. Sinclair’s team demonstrated that SIRT4 was required to shield mitochondria from NAD+-depleting effects of genotoxic stress [21]. A few years ago, Luo et al. discovered that SIRT4 blocked SIRT3-dependent deacetylation of SOD2, thereby reducing SOD2 activity and promoting the increase of mitochondrial ROS [71]. Intriguingly, such effect seemed to be non-enzymatic since a catalytically inactive mutant of SIRT4 also prevented SIRT3-SOD2 interaction [71]. However, in the same year, Shi and co-workers showed that SIRT4 overexpression had an anti-apoptotic effect on glucose-stimulated podocytes via preventing ROS accumulation and the subsequent loss of mitochondrial membrane potential (MMP) [72]. Such contrasting results suggest that SIRT4 elicits context-dependent effects on mitochondrial redox milieu, and this should stimulate future research to better understand how redox-related molecular pathways are regulated by SIRT4.

Like SIRT3, SIRT4 affects mitochondrial dynamics and quality control, even though the precise mechanisms underlying such function remain unclear. SIRT4 has been reported to activate pro-fusion pathways and inhibit mitofission. In fact, the overexpression of SIRT4 was found to inhibit the ERK-mediated phosphorylation of the pro-fission factor DRP1, therefore inhibiting mitochondrial division [73]. In addition, SIRT4 interacts in an enzyme activity-dependent manner with OPA1, leading to elongated mitochondria, as well as to decreased Parkin-associated mitophagy [74]. On this basis, SIRT4 is currently seen as a promising factor that may tip the balance towards hyperfusion, simultaneously reducing the removal efficiency of dysfunctional mitochondria.

To summarize, the literature suggests that via its extremely diverse ADP-ribosyltransferase, lipoamidase and deac(et)ylase activities SIRT4 controls energy homeostasis, metabolic flow in eucaryotic cells. In particular, SIRT4 regulates several cellular metabolic processes, such as glutamine metabolism, FAO and biosynthesis and ATP production. Moreover, convincing evidence pointed to SIRT4 as a key factor controlling mitochondrial dynamics and redox homeostasis.

Among the mtSIRTs, SIRT5 is probably the one investigated most recently, and this is mostly due the absence of particular phenotypes or striking dysmetabolisms found in SIRT5-null mice under basal conditions [75]. Despite its weak deacetylase activity, SIRT5 displays distinct desuccinylase, demalonylase, and deglutarylase activities in the mitochondrial matrix and this is mostly due to the exclusive affinity of SIRT5 for negatively charged acyl-lysine modifications [76,77,78,79,80,81,82]. Many tissues with high metabolic activity (e.g., such as brain and muscle) present high expression levels of SIRT5, thus corroborating the idea that SIRT5 may have an important role in the regulation of energy metabolism [24]. Interestingly, SIRT5 up-regulates ammonia disposal by deacetylating carbamoylophosphate synthetase (CPS1) [77]. This suggested that SIRT5 may serve as a molecular switch to drive metabolic reprogramming and adaptation to changes in nutrient availability (e.g., calorie restriction). Substantial evidence supports the idea that SIRT5 collaborate with SIRT3 to regulate different metabolic pathways, such as OXPHOS and FAO [24]. Interestingly, an extensive reactome analysis performed by Rardin and co-workers demonstrated that the loss of SIRT5 was associated with impaired FAO and reduced ketone body synthesis, and this was linked to the accumulation of hypersuccinylated enoyl-CoA hydratase and 3-hydroxyacyl-CoA dehydrogenase, together with hypersuccinylated HMGCS2 [83]. Later, other researchers found in SIRT5 knock-out mice hundreds of proteins with highly malonylated and glutarylated lysines [84,85]. Noteworthy, increased lysine malonylation seems to be associated with impaired glycolysis, mitochondrial function, and FAO [18,85]. In order to facilitate fatty acyl-CoA degradation, SIRT5 seems to cooperate with SIRT3 to deacylate very long-chain acyl-CoA dehydrogenase (VLCAD), thus promoting its insertion into the inner mitochondrial membrane (IMM) [86]. SIRT5 has also been found to desuccinylate PDH, thus reducing the conversion rate of pyruvate to acetyl-CoA and the entrance of glycolytic carbons into the TCA [87]. Furthermore, a systematic profiling of the mammalian succinylome helped to identify unique desuccinylation sites for SIRT5 and revealed that the knockdown of SIRT5 resulted in an increase of succinylation and inactivation of several mitochondrial proteins, with a consequent impairment of mitochondrial respiration [81]. In addition, Zhang et al. have recently demonstrated that SIRT5 desuccinylates multiple subunits belonging to the ETC complexes and ATP synthase, to promote respiratory chain function [82]. However, some observations seem to suggest that SIRT5 affects the process of stem cell differentiation, and this seems to be linked to its ability to negatively modulate mitochondrial respiration [47]. As seen for other mtSIRTs, the literature suggests that the final consequences of SIRT5 activation on mitochondrial metabolism and oxidative function seems to be influenced by the cellular context and fate.

Recent findings suggested that SIRT5 also serves as a regulator of mitochondrial homeostasis in response to nutrient availability. In particular, SIRT5 modulates mitochondrial dynamics in order to maintain metabolic homeostasis during challenging metabolic conditions. In fact, researchers demonstrated that deletion of SIRT5 led to DRP1 activation and mitochondrial fragmentation. Accordingly, SIRT5 served as mitoprotector by promoting mitochondrial elongation upon starving conditions [88].

SIRT5 seems to be strictly linked also to redox buffering inside and outside mitochondria. In fact, SIRT5 activates IDH2 to produce more NADPH, thus supporting ROS scavenging activities [89]. Besides, SIRT5 increases the activity of glucose 6-phosphate dehydrogenase (G6PD) via deglutarylation, and this enhanced the pentose phosphate pathway (PPP) to ensure the formation of sufficient NADPH for the recycling of oxidized glutathione [89]. In this context, SIRT5 also targets and inactivates pyruvate kinase muscle isozyme 2 (PKM2) via desuccinylation of lysine 498 [90], and this would divert glucose flux into PPP instead of glycolysis, thus leading to more NADPH produced. However, not all the reports agreed on this. Wang and colleagues found that SIRT5 desuccinylates PKM2 at lysine 311 to increase pyruvate kinase activity [91], thus suggesting that SIRT5 might regulate glycolytic flux and NADPH generation in a context-specific fashion and via the regulation of multiple lysine PTMs. Interestingly, SIRT5 also controls the redox environment by increasing the activity of first-line antioxidant enzymes. In 2013, Lin and colleagues observed that SIRT5 desuccinylated SOD1 (a.k.a. cytosolic CuZn-SOD), and this resulted in the increase of the removal of O2.- from the cell [82]. This raised the fascinating hypothesis that SIRT5 may regulate the ROS-scavenging capacity in the mitochondrial intermembrane space, where SOD1 is also present [92]. Moreover, SIRT5 has been recently linked to desuccinylation and inhibition of the peroxisomal acyl-CoA oxidase 1 (ACOX1), which catalyzes the rate-limiting step of FAO-related H_2_O_2_ formation within peroxisomes [93]. On this basis, SIRT5 is today supposed to serve as a possible regulator of redox homeostasis by activating a multiple antioxidant response that involves not only mitochondria, but also other redox-active organelles.

To summarize, even though the precise role of SIRT5 in cell biology is still largely unclear, the results available so far collectively suggest that the SIRT5-dependent PTMs on mitochondrial proteins and enzymes may be critical to mitochondrial function. SIRT5 modulates different pathways that involve glucose oxidation, ketone body formation, fatty acid degradation, ammonia disposal, and redox homeostasis. In addition, SIRT5 seems to serve as key determinant of metabolic rewiring in response to environmental stress, adaptation to variations in accessible nutrients, stem cell differentiation and reprogramming, and this seems to engage not only mitochondria but also other subcellular compartments.

**Figure 2:**
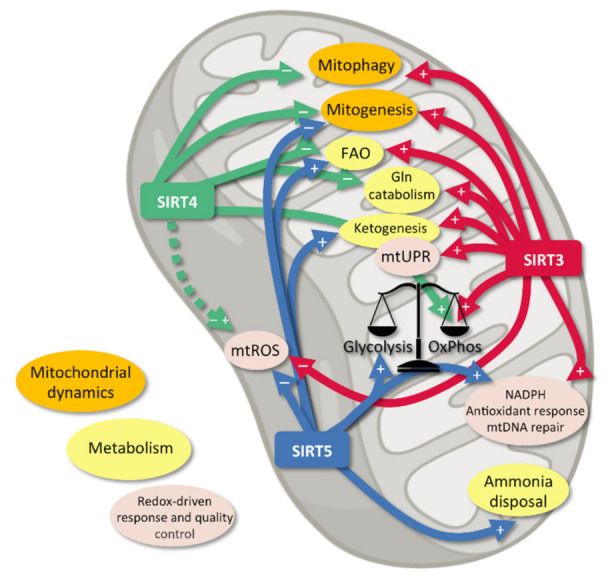
mtSIRT-dependent control of mitochondrial dynamics, metabolism and redox-related response and quality control. FAO, fatty acid oxidation; Gln, glutamine; mtUPR, mitochondrial unfolded protein response. Continued arrows: substantial evidence for effect shown; discontinued arrows: controversial evidence for effects shown; + indicates upregulation, − indicates downregulation.

To sum up, some interlinked pathways underlying the complex crosstalk among all the mtSIRTs can be identified or, in some cases, at least speculated. In some of these pathways mtSIRTs seem to be involved in an antagonistic fashion (e.g., metabolic switch), while other biomolecular routes appear to involve mtSIRTs in a co-operating signalling (e.g., mitochondrial fusion) (Figure 2). mtSIRTs currently represent the core of an emerging research theme, given the strict connection between the mtSIRT-dependent regulation of OS, DNA damage, mitochondrial function and metabolism in the field of aging and disease.

## 3. Sirtuins, Mitochondria and Female Reproduction

**Figure 3:**
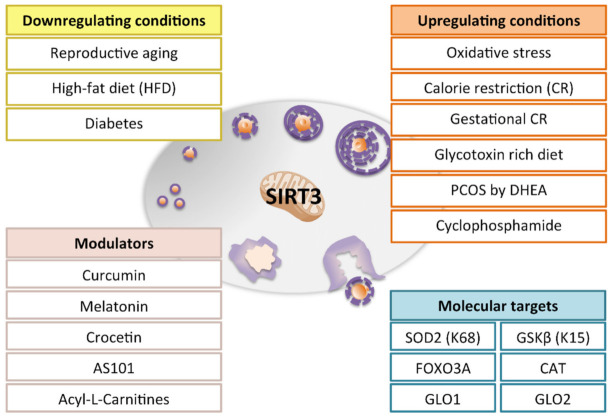
Modulation of SIRT3 in the ovary. The figure shows that SIRT3 expression can be differentially modulated under non-physiological conditions. Reproductive aging, high fat diet and diabetes are conditions that induce a reduction of SIRT3 levels in the ovary. On the other hand, oxidative stress, calorie restriction, gestational calorie restriction, glycotoxins rich diet, PCOS, and chemotherapy (e.g., cyclophosphamide) promote SIRT3 increase, probably favoring an adaptive response. Treatments with beneficial effects on ovarian physiology are associated with restoring of SIRT3 ovarian levels. *C*urcumin and melatonin administration increases SIRT3 expression and ameliorate the aging phenotype. Crocetin or AS101 prevents ovarian SIRT3 up-expression in mice subjected to chemotherapy. Acyl-L-carnitine counteracts SIRT3 increase in ovaries from PCOS mice. Among SIRT3 molecular targets, SOD2, GSK β, FOXO3A, catalase and glyoxalases have been proposed as downstream effectors of SIRT3 in the female gonad.

The role of Sirtuins in female reproductive potential has been clearly demonstrated [94,95]. Nevertheless, information regarding mtSIRTs is mostly limited to SIRT3, with functions that are partially overlapping with SIRT1 (Figure 3).

About ten years ago, the expression of mtSIRTs was demonstrated in mouse and porcine oocytes and in preimplantation embryos [96,97]. In both models, exposure to SIRT-pan inhibitors NAM and Sirtinol induced a reduction of blastocyst formation, in association with increased ROS levels [96,97].

SIRT3 knockdown in oocytes and zygotes determined the establishment of OS, and although meiotic progression was not affected [98], a reduction of blastocyst rate was reported [96]. Embryo developmental arrest was abolished when Sirt3-knockdown zygotes were cultured under low oxygen conditions (mimicking in utero development) [96]. Low blastocyst rate was also observed after parthenogenetic activation of oocytes injected with Sirt3-siRNA [96]. SIRT3 relevance in female germ cell competence for fertilization and early embryo development has been confirmed by observations in human oocytes discarded from IVF program [99], where a positive correlation has been found among Sirt3 mRNA abundance, mitochondrial biogenesis and developmental efficiency from oocytes to blastocyst stage [99]. In mouse preimplantation embryos, increased levels of SIRT3 were observed upon prolonged OS [96].

The fertility potential of Sirt3-null mice is controversial (Table 1). This may be linked to the genetic background of the mouse strains employed in different studies [26,30,96,100]. An analysis of Sirt3 gene single-nucleotide polymorphism (SNPs) in different goat breeds revealed that SIRT3 has a role in reproductive traits and may be useful for the selection of high prolific breeds [101]. Regarding the mouse model, Kawamura et al. [96], observed that oocytes from Sirt3-null mice had a reduced competence for fertilization and blastocyst formation whether Sirt3 was expressed in sperm, suggesting a pivotal role of maternal Sirt3 in early stages of embryo development. As already mentioned, other studies reported that Sirt3-null mice are healthy and fertile at least until the first year of mouse life [26,30,100]. In particular, in a very recent study, Iljas and Homer [100] demonstrated that Sirt3^−/−^ phenotype did not affect ovarian follicle population, with no differences in the number of growing follicles and the number of ovarian oocytes isolated. When focusing on ovarian reserve, the same authors observed that there was an increase in the number of primordial follicles in Sirt3-null mice. In comparison to wild-type female germ cells, Sirt3^−/−^ oocytes showed similar mitochondrial mass, ATP production, and competence to resume meiosis, but higher ROS production, suggesting a decline in mitochondrial function when Sirt3 was abolished. After fertilization similar proportion of preimplantation embryos at blastocyst stage were obtained. Moreover, Sirt3-null females mated with wild-type mice got pregnant and generated healthy and normal-sized offspring.

In early studies, Sirt3-null mice exhibited health issue only when exposed to stressing condition [26,30], such as high fat diet (HFD). HFD in wild type mice induced a drop in oocyte SIRT3 levels, which can be considered the cause of the observed OS [98]. Nevertheless, no differences were observed at ovarian level in HDF Sirt3-null mice [100]. Follicle populations were not affected, and ovarian oocytes completed first meiotic division without spindle and chromosome abnormalities and showed similar rate of ATP and ROS production. This was quite surprising since oocytes contain a very high number of mitochondria, and it would have been reasonable to expect mitochondrial dysfunction upon SIRT3 loss. Interestingly, Sirt3-null oocytes from HFD mice presented increased mitochondrial mass and SIRT1 expression, that authors proposed as a compensatory mechanism [24,100]. Several lines of evidence support the hypothesis that mitochondrial replication can be considered as an attempt of the cell to compensate reduced ATP production from low efficient mitochondria by increasing mitochondrial abundance [100].

Consistently, experiments based on knockin or knockout of oocyte Sirt3 found that this mtSIRT has a pivotal role in counteracting OS reported in HFD and diabetic mice [98,102,103]. This effect seems to be associated with specific deacetylation of SOD2-K68 and GSK3ß-K15, a cytoskeletal regulator essential for the assembly of the meiotic apparatus [98,102,103,104]. Therefore, in addition to ROS scavenging, SIRT3 activity may also influence spindle assembly [98,102,103]. This may be partially ascribed to SIRT3 localization that seems not to be confined in the mitochondria but distributed in the whole cytoplasm as well in GV oocytes [98].

HDF diet and obesity induce epigenetic modifications [105]. Among transgenerational effects, maternal obesity induced a reduction of Sirt3 mRNA in ovaries from female offspring [106]. Although the effects of these molecular alterations on offspring fertility potential have not been investigated, altered redox balance at ovarian level has been associated with the risk of premature ovarian failure [105].

By culturing porcine oocytes in the presence of palmitic acid (PA), Itami et al. [107] developed an in vitro model for the study of obesity-induced dysfunctions. As observed in in vivo experiments, PA oocytes presented a defective phenotype for Sirt3 and mitochondrial dysfunctions [107]. The modulation of AMPK activity by specific activator AICAR or inhibitor dorsomorphin dihydrochloride was demonstrated to restore or worsen, respectively, mitochondrial function and acetylation of mitochondrial proteins in PA-induced obesity oocytes [107]. Similarly, oocyte in vitro culture with melatonin ameliorates obesity-induced phenotype, through the SIRT3-SOD2-dependent mechanism [104].

The administration of a diet with a high content of glycotoxins altered mouse hormonal profile in a way that reminds of early signs of polycystic ovary syndrome (PCOS) [108]. At an ovarian level, these mice exhibited activation of SIRT1-SIRT3 functional network, composed by antiglycative and antioxidant enzymes, such as glyoxalase 1 and 2 (GLO1 and GLO2), SOD2 and CAT, and elements involved in mitochondrial replication and functioning [108].

As it occurs with SIRT1 [94,95], calorie restriction positively influences SIRT3 ovarian expression level with changes to energy metabolism and OS [109]. On the other side, although gestational caloric restriction improved SIRT3 expression and SOD activity in pup ovaries, these molecular mechanisms were not able to counteract the prooxidant conditions of offspring ovarian microenvironment [110].

During reproductive aging, the well-known decline in ovarian reserve is also characterized by a drop in Sirt3 transcript and protein [111,112,113]. Accordingly, age-related decline in Sirt3 mRNA was observed in mouse oocytes [114]. In vivo interventions based on calorie restriction or administration of antioxidant compounds, such as melatonin and curcumin, were proven to ameliorate the aging phenotype through the enhancement of Sirt3 transcript, protein, and activity [111,113,115]. In vitro medium supplementation with quercetin, but not melatonin, restored Sirt3 expression and deacetylation of SOD2 in aged mouse oocytes [114,116].

In post-ovulatory aging, the progressive loss of competence of mature oocytes is accompanied by reduction in Sirt3 levels, increased ROS and mitochondrial dysfunctions [117]. Among laboratory manipulation of gametes, cryopreservation is a condition known to exacerbate post-ovulatory aging [118,119]. As expected, vitrified-thawed mouse oocytes showed a reduction of Sirt3, along with OS markers and mito-damage [120].

Mouse and human granulosa cells (GCs) have the ability to cope with OS by increasing SIRT3, which in turn promotes ROS scavenging by modulating FoxO3a affinity for the promoters of SOD2 and CAT [115,121]. Two studies have investigated the role of SIRT3 in human GCs and cumulus cells (CCs) from IVF patients with different causes for reduced fertility [122,123]. In poor responder women, it has emerged that that Sirt3 expression level is increased and positively correlated with the number of mature oocytes harvested at time of pick-up [122]. By contrast, when Sirt3 gene product, protein, and deacetylation activities were analyzed in GCs and CCs from young women with reduced ovarian reserve or reproductively aged women a reduced SIRT3 function has emerged in comparison to young women with normal ovarian reserve [123].

PCOS is a common endocrine and metabolic disorder, affecting females in their reproductive age with an unclear etiopathogenesis [124]. This condition does not naturally occur in rodents, but several experimental models have been established in order to recapitulate human PCOS features and study underlying molecular mechanisms. Ovaries from a PCOS mouse model induced by dehydroepiandrosterone (DHEA) administration experience prooxidant conditions and are characterized by an adaptive response leading to increased expression of SIRT1 and SIRT3, although a reduction in mitochondrial number was observed [125,126]. As it occurs under other stressing conditions, SIRT3 attempt to cope with altered ovarian microenvironment could be ascribed to/mediated by SOD2 [125,126]. Administration of acyl-L-carnitines in DHEA-induced-PCOS mice promoted mitochondrial replication and reduced glycative stress making unnecessary the activation of SIRT3-SOD2 antioxidant response [126]. Similarly, L-carnitine supplementation in medium of mouse oocytes matured in vitro after a mild oxidative stress prevented Sirt3 rise [127]. By contrast oocytes from PCOS mice obtained after a single injection of estradiol valerate presented a low level of Sirt3 gene expression, that was reverted by in vivo administration of metformin, but not clomiphene citrate [128].

Finally, SIRT3 is an important player of ovarian early adaptive response to gonadotoxic damage induced by cyclophosphamide, in cooperation with SIRT1 [129]. Fertoprotective treatments based on oral administration of natural carotenoid crocetin or synthetic tellurium compound AS101 prevent ovarian damage and SIRT3 increase suggesting that modulation of ovarian microenvironment prior to chemotherapy have beneficial effects for the preservation of ovarian functionality [129].

Regarding SIRT4, very few studies have been carried out in order to establish its role in female fertility. SIRT4-knockout models developed in order to study SIRT4 function in other tissue and organs are fertile [62,130,131]. Nevertheless, Sirt4 expression has been demonstrated to be very high in mature oocytes and zygotes to drop in 2-cell embryos. Sirt4 transcripts remain very low during cleavage and become undetectable at the blastocyst stage [96]. Moreover, in comparison to other mtSIRTs, SIRT4 is the most abundant mtSIRT in the mature oocyte and has a fine-tuned regulation [96,132]. Indeed, unlike SIRT3, SIRT4 overexpression has been associated with detrimental effects on oocyte quality and competence, as demonstrated by reduced ability to complete meiosis, disrupted MII spindle, and altered mitochondrial distribution [132]. The modulation of SIRT4 expression was found to negatively correlate with ATP production and ROS level [132]. Increased SIRT4 was observed in post-ovulatory aged oocytes [133]. Exposure to coenzyme Q10 and/or SIRT4 siRNA was found to attenuate the aging-induced abnormalities including mitochondrial dysfunction, ROS accumulation, spindle abnormalities, and apoptosis in postovulatory aged oocytes by inhibiting SIRT4 increase [133]. Accordingly, in oocytes from aged mice, an up-expression of SIRT4 was detected, and the aged phenotype could be partially restored by SIRT4 knockdown [132].

In human GCs from IVF patients, Sirt4 expression is positively correlated with the number of oocytes retrieved in poor responder patients [122].

Similar to SIRT4, very few studies have been carried out in order to understand SIRT5 role in female reproductive potential. Whole body Sirt5 deletion does not affect the general metabolic and physiological parameters [75], even when combined with Sirt3 deletion [134,135]. Nevertheless, the generation of SIRT5 null phenotypes from mating of male and female heterozygous mice occurred with a low abnormal Mendelian ratio, suggesting a potential role of Sirt5 in embryogenesis or early development [75] that deserves further investigations. Indeed, the expression of Sirt5 was confirmed in mouse oocytes and preimplantation embryos [96]. Mature oocytes and zygotes presented higher levels of Sirt5, while in embryos from 2-cell to blastocyst stage it was detected at low level [96].

Two studies have been focused on SIRT5 role in human granulosa cells. In 2014, Pacella-Ince and coworkers confirmed the presence of Sirt5 transcript, protein and activity in human GCs and CC from IVF patients [136]. They also found out that SIRT5 abundance and activity were decreased in GCs and CCs from women with reduced ovarian reserve or in advanced reproductive age [135]. By contrast, Gonzales-Fernandez et al. [122] observed higher SIRT5 expression in patients aged > 40 years. Moreover, as observed for the other mtSIRTs, these authors observed that Sirt5 expression level was positively correlated with the number oocytes collected from poor responder patients [122].

**Figure 4:**
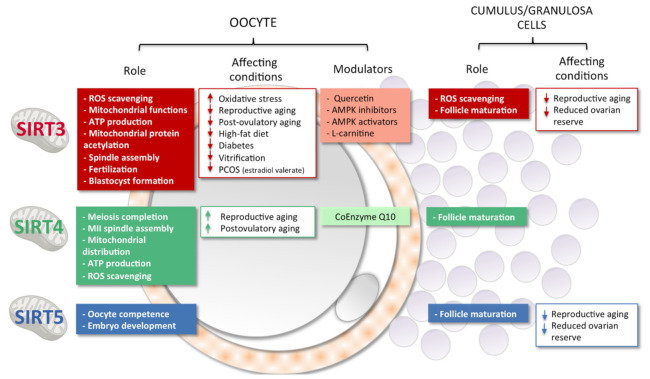
Role of mtSIRTs in oocytes and somatic companions, cumulus cells (CCs) and granulosa cells (GCs) are summarized. Conditions affecting the activity of mtSIRTs and possible beneficial intervention based on media supplementation are listed.

Overall, from the current literature the role of mtSIRTs in the regulation of oogenesis and female reproduction emerges (Figure 4). Based on the results on Sirt3 and Sirt5 knock-out models, these mtSIRTs seems to be involved in some aspects of the reproductive process (i.e., embryo development) that stimulate further research [75,96]. From current literature it clearly emerges that SIRT3 plays a key role in the maintenance homeostasis under stress conditions (i.e., HFD, PCOS and aging) in oocytes, embryos, and ovaries. Interestingly, a peculiar role of SIRT4 in the female reproduction has been revealed [132]. In contrast to other sirtuins, reproductive aging is associated with upregulation of SIRT4 in the female gamete suggesting compensative mechanisms or defective sirtuin regulation.

## 4. Sirtuins, Mitochondria, and Male Reproduction

**Figure 5:**
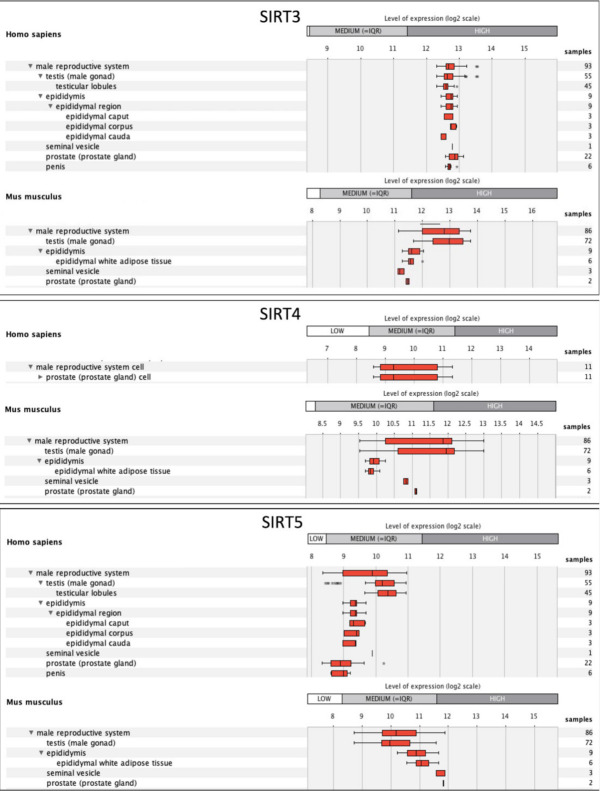
In silico analysis of SIRT3, SIRT4 and SIRT5 gene expression in the male reproductive system using GENEVESTIGATOR software [137] (https://genevestigator.com/gv/index.jsp, access date 28 April 2021). See text for explanation.

The testes are among the adult organs where the high levels of mRNA expression of all sirtuin genes were observed [15]. Since the role of mtSIRTs in male reproduction remains poorly investigated, we have considered relevant to perform an in silico gene expression analysis using GENEVESTIGATOR software [137] (https://genevestigator.com/gv/index.jsp, access date 28 April 2021). The Sirt3, Sirt4, and Sirt5 genes were given as input in the anatomy tool and a data in the form of boxplot-tree were generated (Figure 5). The expression potential represents the normalized expression value for a gene across all experiments available in the database. In the male reproductive system, and more specifically in the testis, Sirt3 is more expressed than Sirt4 and Sirt5 in both humans and mice: Sirt3 is expressed at high level, whereas Sirt4 and Sirt5 are expressed at a medium level. Sirt4 expression is reported to be higher in the mouse than in humans.

Consistent with data above, SIRT3 is the most studied mtSIRT in the male reproductive system. In the testis, it emerges as a component of the SIRT1 network controlling oxidative stress (OS) and a sensor of testis metabolism. Decreased SIRT3 levels were found to promote glycolysis in rat testis [138]. Then, using a pre-diabetic rat model induced by a high-energy-diet, Rato et al. [138] evaluated testicular levels of SIRT3 as a downstream target of PGC-1α, the transcription factor that when deacetylated by SIRT1 activated the transcription of Sirt3 [139]. Both the PGC-1α and SIRT3 protein levels were significantly decreased in testis of pre-diabetic rats. This observation that strongly supported the view that the downregulation of PCG-1α as a consequence of SIRT1 downregulation leads to reduced SIRT3 levels with an amplification of the negative effects of defective glucose metabolism on testicular mitochondria.

A crucial role of SIRT3 in sperm functional electron transport chain and antioxidant defenses has been hypothesized [138]. Fine-tune regulation of mitochondrial metabolism is essential for the spermatogenetic process. The sperm is highly sensitive to oxidative damage due the elevated content of polyunsaturated fatty acid (PUFA) in the membrane and limited antioxidant capacity. Seminal plasma provides the majority of physiologic antioxidant protection against oxidative injury. It is a combination of secretions from the seminal vesicles, prostate, and bulbourethral glands which provide energetic sources, optimal pH, and viscosity for sperm viability and motility. Increased ROS production in the seminal plasma leads to reduced sperm metabolism and motility, resulting in infertility [140]. SIRT3 proteins exist in the seminal plasma [141]. Along with SIRT1, SIRT3 enzymatic activity in seminal plasma was found to be significantly reduced in relation to a high concentration of ROS and abnormal sperm morphology and motility in asthenoteratozoospermia. If sirtuin downregulation is caused by changes in protein levels, post-translational modifications or reduced NAD+ concentration requires further investigation. Consistent with data obtained in seminal plasma are recent findings based on a genome-wide analysis of sperm mRNA content to test for associations with sperm function. LIMMA (linear models for microarray data) analysis identified 20 candidate transcripts as differentially present in low motility sperm, including SIRT3.

A recent study has investigated the role of this sirtuin in the testis damage induced by cadmium (Cd). In a rat model, Wang et al. [142] demonstrated impaired testicular morphology with germ cells loss and vacuoles induced by cadmium exposure induced by autophagy activation. The administration of melatonin, as SIRT3 activator, rescued Cd-induced morphometric injury whereas 3-TYP, a SIRT3 inhibitor, exacerbated Cd effects. When the activities of testicular marker enzymes were tested, they found that activation and inhibition of SIRT3 reduces and worsens, respectively, the negative effects of Cd on the activities of LDH, ACP and AKT [142].

To validate the role of SIRT3, in the same study Sirt3 overexpression model was generated TM3 mouse Leydig cells [142]. The testicular Leydig cell mitochondria are the site of the first enzymatic step in steroidogenesis and hence the disruption of mitochondrial function leads to infertility [143]. The production of testosterone in testicular Leydig cells is essential for the maintenance of spermatogenesis and male fertility. The conversion of testosterone to estradiol is catalyzed by aromatase in steroidogenic tissues and approximately 60% of estradiol in circulation in men is derived from testicular secretion [144]. The increased production of cytokines in inflammatory disease inhibits Leydig cell steroidogenesis and causes male infertility [145]. Based on Ultrastructure analysis revealed that Sirt3 overexpression in Leydig cells protects mitochondria from Cd injury and supporting the hypothesis that SIRT3 mediates the crosstalk between autophagy and apoptosis in testicular Leydig cells.

In addition to toxic environmental compounds, Leydig cells are targeted by bacterial infection in the male reproductive tissue. Several studies have reported that bacterial lipopolysaccharide (LPS) is responsible for impaired testicular steroidogenesis and further effects on fertility [146]. Considering that the early reactions of testicular steroidogenesis occur in mitochondria, Ramatchandirin et al. [147] have investigated the possible role of SIRT4 in LPS-mediated Leydig cell injury. By using the Leydig cell line LC-540 cells, the authors discovered that that downregulation of SIRT4 results in impaired steroidogenesis and decreased cell viability under inflammation. This conclusion was based on the observations that LPS caused mitochondrial dysfunction via the suppression of SIRT4. Moreover, SIRT4 overexpression significantly prevented this effect and increased the mRNA expression of ANT2 supporting the role of SIRT4 in regulating ATP homeostasis. The overexpression of SIRT4 also upregulated the expression of LRH-1, StAR, P450scc, 3β-HSD, and 17β-HSD in LPS treated LC-540 cells. Finally, the increased viability associated with the overexpression of SIRT4 in LPS treated cells was ascribed to the prevention of the mitochondrial apoptotic pathway.

In 2015, Sirt5 was reported to be expressed in the cytosol of mouse spermatogonia, spermatocyte, and round spermatid [148]. Based on the control of CPS1 by SIRT5, [15,77], this sirtuin was able to play a role in the regulation of mitochondria and physiological stress during spermatogenesis by acting as a regulator of urea cycle and detoxification.

**Figure 6:**
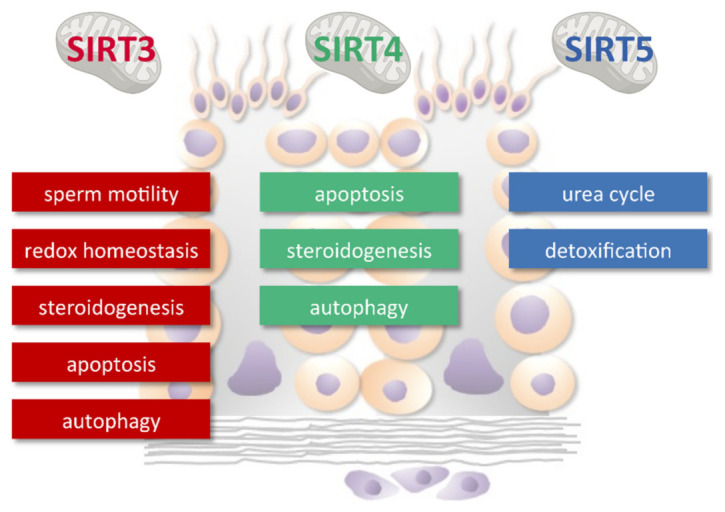
Main functions of mtSIRTs in spermatogenesis and male reproduction. MtSIRTs participate in homeostasis maintenance under stress conditions.

Based on the above results and considering that knock-out models generated so far do not display effects on male fertility [62,75,96], we can speculate that mtSIRTs operate in a complex network that participate in homeostasis maintenance under stress conditions rather than in the in physiological regulation of spermatogenesis and male reproduction (Figure 6). Sirt3 and Sirt4 are involved in the response to metabolic dysfunctions, autophagy, and apoptosis in the testis. The studies in the semen and sperm reveal the role of Sirt3 in the dysregulation of the redox milieu in sperm with reduced motility. The studies in Leydig cells reveal the role of Sirt3 and Sirt4 in regulating testicular steroidogenesis. Although the expression of SIRT5 in germ cells has been clearly shown, its role deserves further investigation.

## 5. Dietary and Pharmacological Interventions Modulating Mitochondrial Sirtuins

**Figure 7:**
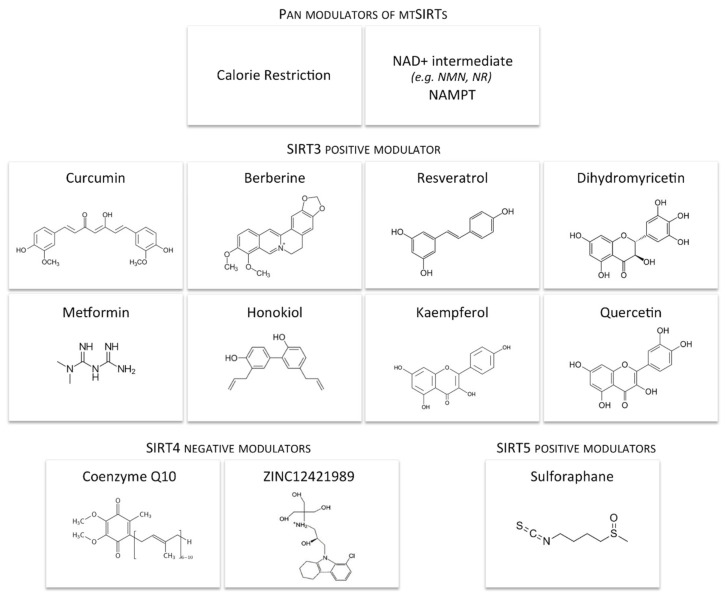
Dietary and pharmacological interventions alleviating manifestations of many diseases by modulating mtSIRTs.

Based on basic research and pre-clinical studies, the modulation of mtSIRTs may be helpful for the improvement of fertility potential. In this regard, here, we report current knowledge about potential regulators of mtSIRTs (Figure 7), taking into account that biochemical activators of mtSIRT are not available.

Studies on the mouse model have shown that mtSIRT modulation may have a beneficial effect on health, alleviating manifestations of many diseases, including diabetes, metabolic syndrome, cardiomyopathies, non-alcoholic hepatic steatosis, hyperinsulinism-induced dyslipidemia, chronic inflammation, neurodegenerative diseases, and some types of cancer [149].

The conservation or restoration of the cellular and mitochondrial pool of NAD+ may represent a promising strategy to boost sirtuin activity. NAD+ intermediate supplementation such as NMNs appears to restore NAD+ levels in both nuclear and mitochondrial compartments of cells. [20]. Another NAD+ intermediate, nicotinamide riboside (NR), can be converted to NAD+, after conversion to NMN via NR kinase (Nrk) [20]. NR supplementation increases mitochondrial NAD+ levels and stimulates SIRT3-mediated deacetylation of mitochondrial proteins [150].

Nicotinamide phosphoribosyltransferase (NAMPT) converts nicotinamide (NAM) to nicotinamide mononucleotide, which is converted to NAD+ by nicotinamide adenylyltransferase in the mammalian biosynthetic pathway. NAMPT is a rate limiting enzyme in the conversion of NAM to NAD+ and may thus alter the NAD+/NADH ratio, which is crucial for the activation and regulation of SIRT3 transcription [151]. Recently, NAMPT has been reported to increase the levels of SIRTs and to have cell-protective activities [152].

Moreover, a number of currently available drugs and nutraceuticals have been demonstrated to activate SIRTs directly or via allosteric activation. Honokiol, a natural biphenolic compound derived from the bark of Magnolia trees, is present in the mitochondrion where enhances SIRT3 expression and binds SIRT3 to increase its activity [153].

Metformin, a biguanide derivative, is the first-line drug for the treatment of type 2 diabetes. Moreover, it is also employed in the PCOS, due to its insulin sensitizer activity [154]. Metformin increases SIRT3 expression and activity [128]. Metformin has been shown to promote phosphorylation of AMPK, which leads to protection against oxidative injuries [155]. Comparing the effects of metformin and clomiphene citrate on the expression of the Sirt3 gene in oocytes obtained from mice induced by PCOS, it was found that both treatment regimens returned to the baseline values the altered parameters. The gene of Sirt3 was significantly reduced in the PCOS group compared to the control. Also, no significant difference was found in the expression of Sirt3 between clomiphene and PCOS group, whereas, in the metformin group, Sirt3 expression had a higher rate of expression in comparison with the PCOS group. Thus, it was been showed that metformin is capable of preventing the downregulation of the Sirt3 gene in oocytes from PCOS mice [128].

Resveratrol (3,5,4′-trihydroxy-trans-stilbene), a polyphenol found in natural products including grapes [156], is the most powerful natural sirtuin activator. Mimicking the positive effect of caloric restriction, resveratrol helps in the treatment or prevention of obesity and preventing reproductive aging in the mouse model. The deacetylation of the master regulator of mitochondrial PGC-1α by SIRT1 leads to upregulation of SIRT3 [157].

Quercetin, a flavonoid present in vegetables, fruits, herbs, and red wine, [158] reduces ROS via SIRT3-mediated acetylation of SOD2 and improves oocyte quality in reproductively aged mice [116]. Recently, quercetin has been extensively investigated as a therapeutic option in patients with PCOS. Treatment with quercetin improved the PCOS related disturbances in estrous cycle, lipid profile, serum levels of testosterone, estradiol and progesterone, and IR. Besides, the expression levels of AMPK and SIRT1 in ovarian tissue were upregulated in the rats, which received quercetin. Quercetin also reversed the PCOS-induced alteration in adipose tissue levels of adiponectin, visfatin, and resistin. Modulation of energy homeostasis through key components involved in this axis, as well as the regulation of hormones releasing from adipose tissue may be the main underlying mechanisms for positive effects of quercetin in PCOS [159].

Berberine is a yellow alkaloid mainly isolated from the Chinese herb Coptis chinensis. Its hypoglycemic effect is similar to that of metformin [160]. Glucose, insulin, lipid profiles, and hepatic OS parameters was improved after BER-chloride administration in HFD mice. Further, berberine-chloride improved transaminases enzymes, pro-oxidant, and antioxidant defense system, PI3K, AKT, and PTEN by SIRT1 and SIRT3 activation in the liver [161,162,163]. In PCOS patients, berberine improves insulin sensitivity and ovulation function, however, the mechanism by which berberine initiates glucose metabolism-related signaling pathways in ovarian cells remains unknown [164]. A recent in vitro study has unveiled a new mechanism by which berberine promotes ovarian cell glucose uptake and demonstrated that SIRT3 ubiquitination is involved in the insulin sensitizing effect of berberine [165].

Curcumin, a polyphenol extract of *Curcuma longa*, is an antioxidant and anti-inflammatory agent known to prevent ovarian aging. In female mice, curcumin treatment resulted in increased ovarian volume and number of follicles and was associated with elevated anti-Müllerian hormone and estrogen and diminished FSH serum levels. Furthermore, enhanced oocyte maturation, fertilization, and embryo development plus reduced OS were seen in the curcumin group. Also, the expression of Gdf9, Bmp15, Sirt1, and Sirt3 genes was increased in the curcumin group [113].

Dihydromyricetin (DHM) is a natural flavonol with a wide range of health benefits including anti-inflammatory, antitumor, and antioxidant effects. DHM protective effect is dependent on activation of the PGC-1α/SIRT3 signaling [166].

Recently, docking studies revealed that kaempferol and sulforaphane, and exhibits the highest docking scores against SIRT3 and SIRT5, respectively [167].

Generally, a specific inhibitor, which may be a considerable candidate for the terapeutic treatment, is more difficult to develop than an activator. A recent work proposed ZINC12421989 as a potential inhibitor of SIRT4 [168]. Further functional study is needed to validate the inhibitory effect of ZINC12421989 on SIRT4 protein and subsequent biological phenotypes.

In addition to drug supplement, nutrient status regulates the activation of mtSIRTs. Controlling nutrient intake may be a more mild and suitable manner to control mtSIRTs. CR is a dietary regimen that offers benefits by improving mitochondrial function and quantity control, and subsequently [169]. Long-term CR can up-regulate the level of SIRT3 while has an opposing effect on SIRT4 expression [170]. SIRT3 mediates the reduction in oxidative damage and prevents age-related hearing loss under CR [171]. Additionally, SIRT4 is involved in CR-mediated potentiation of amino acid-stimulated insulin secretion [62]. Therefore, the benefits of CR may also, at least in part, rely on the regulation of mtSIRTs. However, further evidence is needed to validate this hypothesis.

## 6. Conclusions and Future Remarks

Overall, from the current literature the role of mtSIRTs in the regulation female and male reproduction emerges. Although this is not surprising, attention from researchers on mtSIRTs has been increasing only in last years, in contrast to other sirtuins. The majority of the work is based on in vitro and in vivo studies on animal models and very few information has been obtained on humans. Moreover, the latter are based on isolated cells from IVF patients, while clinical studies are missing. Most the works have focused on SIRT3 with a prevalence of studies on female reproduction. Numerous studies have provided evidence that SIRT3 improves the competence of oocytes in humans and animal models. Moreover, SIRT3 protects the oocyte and early embryos against stress conditions. The relationship between derangement of SIRT3 signaling and imbalance of ROS and antioxidant defenses in testes has also been demonstrated. Studies on SIRT4 and SIRT5 remain very limited and do not allow for an understanding of their physiological role in the reproductive system. On this basis, present review provides challenges and opportunities to stimulate research on the role of mtSIRTs in mammalian oocytes, sperm, embryos, ovaries, and testes and opens up new frontiers in the establishment of mtSIRT-based therapeutic applications in female and male infertility. In the future, the mtSIRT interactome may serve as a roadmap to deepen our understanding of mtSIRT biology and may facilitate the uncovering of additional mechanisms that control sirtuin function during cellular homeostasis and stress.

## Figures and Tables

**Table 1:** Impact of mtSIRT knockout on fertility potential and offspring health.

Mouse	Genotype	Gender	Observations	Reference
Sirt3^Gt(neo)218Lex^ C57BL6	Sirt3^−/−^	female	Reduced fertilization and blastocyst rate when oocytes from Sirt3^−/−^ mice were employed in IVF.	[96]
		male	No effects when sperm from Sirt3^−/−^ mice were employed in IVF.	
129-SIRT^tm1.1Fwa^/J	Sirt3^−/−^	female	Increased primordial follicles. Increased ROS in oocytes. No effects on fertility.	[100]
Sirt5^floxed^/CMV-Cre	Sirt5^−/−^	female and male	Loss of approximately 40% of Sirt5^−/^^−^ offspring generated from heterozigous Sirt5^+/^^−^ mice. Surviving Sirt5^−/−^ pups appeared normal.	[75]
Sirt3^−/−^ Sirt5^−/−^ C57BL/6J	Sirt3^−/−^ Sirt5^−/−^	female and male	Normal fertility, litter size, female/male sex ratio and adult development. No gross abnormalities were revealed after autoptic analysis.	[136]

## Abbreviations

17β-HSD	17β-hydroxysteroid dehydrogenase
3β-HSD	3β-hydroxysteroid dehydrogenase
AceCS2	acetyl-CoA synthetase 2
ACOX1	acyl-CoA oxidase 1
ADP	adenosine diphosphate
AKT	protein kinase B
AMPK	5′ AMP-activated protein kinase
ANT2	ATP/ADP translocase 2
ATP	adenosine triphosphate
BMP15	bone morphogenetic protein 15
CAT	catalase
CCs	cumulus cells
Cd	cadmium
CPS1	carbamoylophosphate synthetase
CR	calorie restriction
DHEA	dehydroepiandrosterone
DHM	dihydromyricetin
DNA	deoxyribonucleic acid
DRP1	dynamin-related protein 1
ERK	extracellular signal-regulated kinase
ETC	electron transport chain
FAO	β-oxidation of fatty acids
FOXO3A	forkhead box O3 a
G6PD	glucose 6-phosphate dehydrogenase
GCs	granulosa cells
GDF9	growth differentiation factor 9
GDH	glutamate dehydrogenase
GLO	glyoxalase
GSH	glutathione
H_2_O_2_	hydrogen peroxide
HFD	high fat diet
HMGCS2	3-hydroxy-3-methylglutaryl CoA synthase-2
ICDH2	isocitrate dehydrogenase 2
IMM	inner mitochondrial membrane
IVF	in vitro fertilization
LCAD	long-chain acyl-CoA dehydrogenase
LPS	lipopolysaccharides
LRH-1	liver receptor homolog-1
MCD	malonyl CoA decarboxylase
MFN2	mitofusin 2
MMP	mitochondrial membrane potential
mRNA	messenger ribonucleic acid
mtDNA	mitochondrial DNA
MTPα	mitochondrial trifunctional protein α-subunit
mtQC	mitochondrial quality control
mtROS	mitochondrial reactive oxygen species
mtSIRT	mitochondrial sirtuin
mtUPR	mitochondrial unfolded protein response
NAD	nicotinamide adenine dinucleotide
NADP	nicotinamide adenine dinucleotide phosphate
NAM	nicotinamide
NAMPT	nicotinamide phosphoribosyltransferase
NMN	nicotinamide mononucleotide
NR	nicotinamide riboside
OGG1	8-oxoguanine-DNA glycosylase 1
OPA1	protein GTPase optic atrophy 1 protein
OS	oxidative stress
OXPHOS	oxidative phosphorilation
P450scc	cholesterol side-chain cleavage enzyme
PA	palmitic acid
PCOS	polycystic ovary syndrome
PDH	pyruvate dehydrogenase
PGC-1α	peroxisome proliferator-activated receptor gamma coactivator 1-alpha
PI3K	phosphoinositide 3-kinase
PKM2	pyruvate kinase muscle isozyme 2
PPP	pentose phosphate pathway
PTEN	phosphatase and tensin homolog
PTM	post-translational modification
PUFA	polyunsaturated fatty acid
ROS	reactive oxygen species
siRNA	small interfering RNA
SIRT	sirtuin
SNP	single-nucleotide polymorphism
SOD2	manganese-dependent superoxide dismutase
StAR	steroidogenic acute regulatory
TCA	tricarboxylic acid cycle
VLCAD	very long-chain acyl-CoA dehydrogenase
